# Adipose Tissue Fatty Acid Patterns and Changes in Anthropometry: A Cohort Study

**DOI:** 10.1371/journal.pone.0022587

**Published:** 2011-07-21

**Authors:** Christina Catherine Dahm, Anders Gorst-Rasmussen, Marianne Uhre Jakobsen, Erik Berg Schmidt, Anne Tjønneland, Thorkild I. A. Sørensen, Kim Overvad

**Affiliations:** 1 Department of Cardiology, Center for Cardiovascular Research, Aalborg Hospital, Aarhus University Hospital, Aalborg, Denmark; 2 Department of Epidemiology, School of Public Health, Aarhus University, Aarhus, Denmark; 3 Department of Mathematical Sciences, Aalborg University, Aalborg, Denmark; 4 Institute of Cancer Epidemiology, Danish Cancer Society, Copenhagen, Denmark; 5 Institute of Preventive Medicine, Copenhagen University Hospitals, Copenhagen, Denmark; Fundação Oswaldo Cruz, Brazil

## Abstract

**Introduction:**

Diets rich in n-3 long chain polyunsaturated fatty acids (LC-PUFA), but low in n-6 LC-PUFA and 18:1 *trans*-fatty acids (TFA), may lower the risk of overweight and obesity. These fatty acids have often been investigated individually. We explored associations between global patterns in adipose tissue fatty acids and changes in anthropometry.

**Methods:**

34 fatty acid species from adipose tissue biopsies were determined in a random sample of 1100 men and women from a Danish cohort study. We used sex-specific principal component analysis and multiple linear regression to investigate the associations of adipose tissue fatty acid patterns with changes in weight, waist circumference (WC), and WC controlled for changes in body mass index (WC_BMI_), adjusting for confounders.

**Results:**

7 principal components were extracted for each sex, explaining 77.6% and 78.3% of fatty acid variation in men and women, respectively. Fatty acid patterns with high levels of TFA tended to be positively associated with changes in weight and WC for both sexes. Patterns with high levels of n-6 LC-PUFA tended to be negatively associated with changes in weight and WC in men, and positively associated in women. Associations with patterns with high levels of n-3 LC-PUFA were dependent on the context of the rest of the fatty acid pattern.

**Conclusions:**

Adipose tissue fatty acid patterns with high levels of TFA may be linked to weight gain, but patterns with high n-3 LC-PUFA did not appear to be linked to weight loss. Associations depended on characteristics of the rest of the pattern.

## Introduction

Overweight and obesity are growing health problems, with implications for risk of diseases such as diabetes, cardiovascular disease and some cancers [1]. In large epidemiological studies, a common measure of general obesity is body-mass index (BMI), which shows strong associations with mortality [2]. Waist circumference (WC) has been used as a measure of abdominal adiposity [3], and is predictive of mortality [2], [4], while WC controlled for BMI (WC_BMI_) has been used as a proxy for visceral fat [4]–[6], believed to be a particularly strong risk factor for cardiovascular morbidity and mortality [4]. Determinants of weight gain are likely to be persistent, leading to risk of obesity, and increases in abdominal adiposity are related to greater risk of metabolic disease [7].

The influence on weight gain of specific nutrients such as types of fat beyond their caloric value is unclear [8]–[11]. Animal models indicate that intake of n-3 long chain polyunsaturated fatty acids (LC-PUFA), or of conjugated linoleic acids (CLA), reduces body fat accumulation, while intake of n-6 PUFA, such as 18:2 n-6 or 18:1 *trans*- fatty acids (TFA) increases body fat accumulation, but evidence in humans is limited [12]–[15]. Effects may be due to altered gene expression, or to the inflammatory eicosanoid compounds that are derived from n-3 and n-6 PUFA [12], but other mechanisms may also operate [11], [13], [16].

The composition of adipose tissue reflects medium to long term dietary fatty acid intake as well as the influence of genes, metabolism, lifestyle and the intake of other nutrients [17], [18]. Investigation of the associations between individual adipose tissue fatty acids and body size is complex as fatty acids are highly correlated through common dietary sources and metabolic processes. Associations may also be weak when exposures to single fatty acids are considered in isolation and adjusted for correlated confounders. As adipose tissue is composed of families of fatty acids that share the same metabolic pathways, it is likely that the patterns of fatty acids present in tissues may provide information on the systemic effects of fat types, as well as provide new hypotheses regarding potential health effects of other fatty acid families than the commonly studied TFA, n-3 or n-6 PUFA. The correlation structure among a large number of exposure variables, such as the fatty acid profile, may be characterized by principal component analysis (PCA) [19], [20], which extracts global patterns among variables. Often used to investigate dietary patterns [19], previous studies using PCA have shown that serum fatty acid patterns are predictive of cardiovascular disease risk [21] and of development of the metabolic syndrome [22]. However, patterns may be complex, and often only those patterns that are amenable to interpretation are reported [19], which constrains the hypothesis-generating nature of exploratory data analysis.

In this study, we aimed to prospectively investigate associations between all patterns of adipose tissue fatty acids fulfilling a priori defined statistical criteria and changes in weight, WC, and WC_BMI_.

## Methods

### Study population

Diet, Cancer and Health is a Danish cohort study [23] that in 1993-97 recruited 27 179 men and 29 876 women, aged 50–64, from the urban areas of Copenhagen or Aarhus, Denmark. All participants gave written informed consent, and the study was approved by both the Copenhagen and the Aarhus Ethical Committees, as well as the Danish Data Protection Agency. A sample of 1869 cohort members was drawn for the present study using simple random sampling. Participants were subsequently excluded from this analysis if they had a baseline or follow-up diagnosis of vascular disease, diabetes mellitus, cancer, or COPD, if they did not participate in the follow-up data collection, or if fatty acid data were completely missing.

### Data collection

At recruitment, participants completed questionnaires on lifestyle and medical history, including leisure time physical activity [24], [25]. The questionnaires were checked by trained interviewers during a clinical visit. At this visit staff measured participant height, weight, and WC, and a biopsy of adipose tissue was taken from the buttock. Participant height was measured to the nearest half centimetre when standing without shoes. Weight was measured to the nearest 0.1 kg using digital scales, with participants wearing only light clothing or underwear. WC was recorded to the nearest 0.5 centimetre and was measured using a rigid measuring tape at the narrowest point between the lower rib and the iliac crest while standing. In cases of indeterminate waist narrowing, waist circumference was measured half way between the lower rib and the iliac crest. The biopsy was taken as previously described [26], using a luer-lock system (Terumo, Terumo Corporation, Tokyo) consisting of a needle, a venoject multisample luer adaptor, and an evacuated blood tube. Samples were subsequently flushed with nitrogen and stored at −150°C until analysis.

In 1999–2002 follow-up questionnaires and a rigid measuring tape were sent to all participants. Follow-up weight and WC were measured by participants at home and recorded in the follow up questionnaires. Participants were asked to measure their WC at the level of the umbilicus to simplify interpretation. Adipose tissue was not sampled during this data collection.

### Fatty acid determination

For the random sample of 1869 cohort members, the biopsies were thawed and 2–4 mg adipose tissue were removed and analysed as previously described [18] After *trans*-esterification by potassium hydroxide in methanol, fatty acid composition was determined by gas chromatography on a CP-sil 88 60 m×0,25 mm ID capillary column, consisting of a highly substituted, stabilized cyanopropyl stationary phase, using a Varian 3900 GC with a CP-8400 auto sampler (Varian, Middleburg, The Netherlands) equipped with a flame ionization detector. The carrier gas was helium. Individual fatty acids were identified using commercially available standards (Nu-chek-Prep, Inc., Minnesota, USA). This approach allows quantification of fatty acid methyl esters of 12 to 22 carbon atoms, and the results are expressed as percentages of total fatty acids. 34 fatty acids were determined (Table 1). Peaks for 18:1n-6t and 18:1n-8t could not be separated, nor could those for 18:1n-10t and 18:1n-12t, and the combined fatty acid proportions are analysed here. Eight fatty acids had missing values for some participants (n = 1–10 for 18:1n-6t+8t, 18:1n-9t, 18:1n-10t+12t, 18:1n-7t, 20:4n-3, and 22:6n-3, n = 26 for 18:3n-6 and n = 47 for 16:1n-7t). The interassay coefficients of variation ranged from 0.7% to 11.7%.

**Table 1:** Nomenclature and names of fatty acids.

Class	Nomenclature	Common name
Saturated fatty acids (SFA)		
	12:0	Lauric acid
	14:0	Myristic acid
	15:0	Pentadecanoic acid
	16:0	Palmitic acid
	17:0	Heptadecanoic acid
	18:0	Stearic acid
	19:0	Nonadecanoic acid
	20:0	Arachidic acid
Monounsaturated fatty acids (MUFA)		
	14:1n-5	Myristoleic acid
	16:1n-7	Palmitoleic acid
	18:1n-7	Asclepic acid
	18:1n-9	Oleic acid
	20:1n-9	n-9 Eicosenoic acid
	20:1n-11	n-11 Eicosenoic acid
	22:1n-9	Erucic acid
*trans-*fatty acids (TFA)		
	16:1n-7 (Δ9t)	*trans-*Palmitoleic acid
	18:1n-10+12 (Δ6t+Δ8t)*	
	18:1n-9 (Δ9t)	Elaidic acid
	18:1n-6+8 (Δ10t+Δ12t)*	
	18:1n-7 (Δ11t)	Vaccenic acid
	18:2n-6 (Δ9c12t)	cis-trans octadecadienoic
	18:2n-6 (Δ9t12c)	trans-cis octadecadienoic
	18:2n-6 (Δ9c11t)	Rumenic acid
Polyunsaturated fatty acids (PUFA)		
	18:2n-6	Linoleic acid
	18:3n-3	α-Linolenic acid
	18:3n-6	γ-Linolenic acid
	20:2n-6	Eicosadienoic acid
	20:3n-6	Dihomo-γ-linolenic acid
	20:4n-3	Eicosatrienoic acid
	20:4n-6	Arachidonic acid
	20:5n-3	Eicosapentaenoic acid
	22:4n-6	Docosatetraenoic acid
	22:5n-3	Docosapentaenoic acid
	22:6n-3	Docosahexaenoic acid

*Peaks for 18:1n-10t and 18:1n-12t, and for18:1n-6t and 18:1n-8t, could not be separated.

**Δ nomenclature in parentheses indicates the location of the double bond from the carboxyl terminal.

### Anthropometric measures

Baseline BMI was calculated as weight (kg)/height^2^ (m^2^), and follow-up BMI was determined using follow-up weight. WC_BMI_ was defined as the residual values from the sex-specific regression equations of WC on BMI, using baseline or follow-up WC and BMI values as appropriate. Changes in weight (kg/yr), WC (cm/yr) or WC_BMI_ (cm/yr) were calculated as mean annual changes (follow-up measure – baseline measure/number of years between measurements).

### Statistical analysis

Missing values in the fatty acid data were imputed using an expectation maximization (EM) algorithm [27]. Briefly, entries corresponding to missing values are initially replaced by the variable mean. For each variable, these initial imputations are subsequently refined by replacing them with predicted values based on a linear regression of the subset of completely observed variable values on the remaining 33 fatty acid variables. After one complete sweep through all variables, the scheme is iterated until differences in imputed values are no more than 0.0001. Finally, the missing values in the dataset are replaced by the imputed values [28].

To determine fatty acid patterns in adipose tissue, we conducted sex-specific PCA on the correlation matrix of the 34 untransformed fatty acid variables, expressed as percentages of total fatty acids. This is equivalent to analysis of the covariance matrix of standardized variables, and avoids undue influence of fatty acids with great variation. Histograms of the distributions of the 34 fatty acid variables overlaid with the appropriate normal curve were visually inspected to verify approximate normality. PCA is a dimension reduction technique that aims to explain the variation observed within the data by constructing linear combinations of the entered variables, termed principal components [29]. The weights used to construct the linear combinations, referred to as factor loadings, make up the principal components (PC). These weights indicate the correlation of each fatty acid to the PC in question and the loadings are used to interpret the PC. All individuals were assigned PC scores on a continuous scale according to where their standardized fatty acid profiles fell compared to the PC in question. PCs are orthogonal to one another, and their scores are uncorrelated. To be retained for further analysis as described below, PCs had to have an eigenvalue ≥1.25 [30], and pass visual inspection of a scree plot (PCs plotted against cumulative explained variance) [31].

### Regression analysis

For both men and women, the scores of retained PCs, expressed per standard deviation, were entered as continuous variables into separate multiple linear regression analyses of changes in weight, WC or WC_BMI_, adjusted for age (yrs; continuous) and physical activity (<3.5 /≥3.5 hrs per week; binary). A validation study within this cohort has shown that due to associations with baseline anthropometry, analysis of changes in WC in this dataset should be adjusted for baseline BMI and WC [32]. The models were as follows: Change in weight  =  PC score + age + physical activity + baseline height + baseline weight. Change in WC  =  PC score + age + physical activity + baseline height + baseline weight + baseline WC. Change in WC_BMI_  =  PC score + age + physical activity + baseline height + baseline weight + baseline WC. Baseline height, weight and WC were all continuous variables. These analyses were investigated for linearity using augmented component plus residual plots of the adjusted regression models [33].

All analyses were conducted using Stata 11 (StataCorp LP, College Station, Texas). 95% confidence intervals that did not include zero were considered statistically significant.

### Assessment of pattern reproducibility

To assess the reproducibility of the PCA results, men and women were divided randomly into two groups. Sex-specific PCA was performed in each group, PCA1 in group 1 and PCA2 in group 2. Scores were generated by applying factor loadings from PCA1 and PCA2 to group 1. Reproducibility between PCA1 and PCA2 was assessed by calculating Pearson's correlation coefficients between scores in group 1. Factor loadings from PCA1 and PCA2 were then applied to group 2, and Pearson's correlation coefficients were calculated in group 2. The average of these correlations can be interpreted as a measure of the stability of PCA in these data [34].

## Results

**Figure 1:**
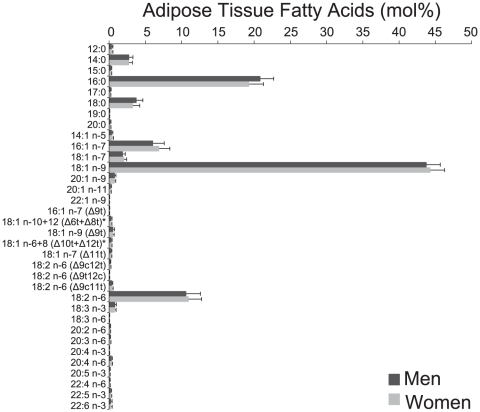
Adipose tissue fatty acid composition in 556 men and 544 women. Data are expressed as mean (mol%) and error bars represent SD. Δ nomenclature in parentheses indicates the location of the double bond from the carboxyl terminal. * Peaks for 18:1n-10t and 18:1n-12t, and for18:1n-6t and 18:1n-8t, could not be separated.

Participants were excluded from the study if they had a baseline diagnosis of vascular disease (n = 124), diabetes mellitus (n = 11), cancer (n = 14), or chronic obstructive pulmonary disease (n = 4), if they did not participate in the follow-up data collection (n = 337) or if fatty acid data were completely missing (n = 71). Of these 1258 participants, 158 were diagnosed with vascular disease, diabetes, cancer or chronic obstructive pulmonary disease during follow-up. These participants were excluded as disease may alter body size determined at follow-up, leaving 1100 participants for analysis. Median follow-up time was 5.4 years. Characteristics of the study participants are shown in Table 2. Only 15-20 EM iterations were necessary to stabilize imputed missing fatty acid values. Mean fatty acid proportions for men and women, including imputed values, are shown in Figure 1. Many fatty acids were highly correlated (Figure S1).

**Table 2:** Baseline characteristics of 1100 participants with baseline adipose tissue biopsies and follow up data.

		Men (n = 556)	Women (n = 544)
Age (yrs)		55.6 (50.8–64.1)	55.5 (50.7–63.7)
Height (cm)		176 (166–188)	165 (154–174)
Weight (kg)		81.1 (66.3–103.9)	66.2 (52.9–87.9)
Baseline BMI (kg/m^2^)		26.2 (22.0–31.8)	24.3 (19.9–32.3)
Follow-up BMI (kg/m^2^)		26.2 (22.0–32.1)	24.3 (19.8–32.4)
Annual change in weight (kg/yr)		0.1 (−1.4–1.3)	−0.1 (−1.4–1.1)
WC (cm)		94.5 (82.0–111.0)	79.0 (67.0–101.0)
Follow-up WC (cm)		98.0 (85.0–114.0)	86.0 (71.0–110.0)
Annual change in WC (cm/yr)		0.6 (−1.3–2.4)	1.2 (−0.9–3.6)
Physical activity (%)			
	<3.5 hrs/wk	61.8	58.9
	>3.5 hrs/wk	38.2	41.8

Data are medians (5^th^ – 95^th^ percentile) or percentages as appropriate.

Assessment of pattern stability indicated good reproducibility between PCA1 and PCA2, with absolute Pearson correlation coefficients generally >0.8 (data not shown).

**Figure 2:**
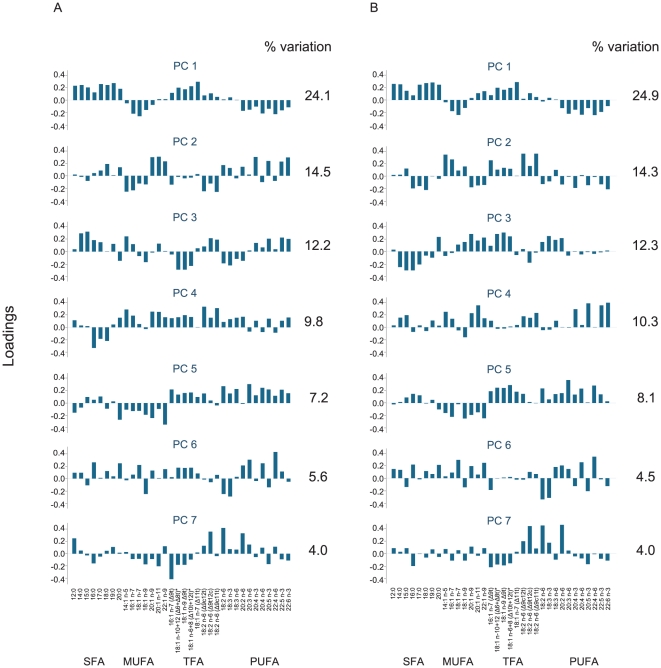
Loading plots for 7 (A) male and (B) female principal components (PC). Y axes indicate factor loadings. SFA  =  saturated fatty acids, MUFA  =  monounsaturated fatty acids, TFA  =  *trans-*fatty acids, PUFA  =  polyunsaturated fatty acids. Δ nomenclature in parentheses indicates the location of the double bond from the carboxyl terminal. * Peaks for 18:1n-10t and 18:1n-12t, and for18:1n-6t and 18:1n-8t, could not be separated.

When conducting sex-specific PCA on the entire study sample, similar patterns in highly loading fatty acids were seen for men and women across several of the 7 sex-specific components retained (explaining a total of 77.6% and 78.3% of the variance respectively, Figure 2, Table S1). All PCs retained for further analysis showed linear relationships with changes in weight, WC and WC_BMI_ when adjusted regression models were investigated using augmented component plus residual plots (data not shown). The results of these linear regression models are therefore presented here.

Both male PC1 and female PC1 were characterized by positive loadings of saturated fatty acids (SFA) and TFA, while most monounsaturated fatty acids (MUFA) and LC-PUFA loaded negatively (Figure 2). There were no associations between PC1 and changes in body size (Table 3).

**Table 3:** Regression coefficients (95% CIs) of regressions of annual change in weight (kg/yr), annual change in WC (cm/yr), and annual change in WC_BMI_ (cm/yr) on SD of principal component (PC) scores 1-7 in men and in women.

	Change in weight^1^		Change in WC^2^		Change in WC for change in BMI^3^	
Exposure	Regression coefficient	P value	Regression coefficient	P value	Regression coefficient	P value
**MEN**						
PC1	−0.01 (−0.09; 0.06)	0.723	−0.03 (−0.14; 0.07)	0.516	−0.03 (−0.11; 0.06)	0.551
PC2	0.09 (0.02; 0.15)	0.013	0.02 (−0.08; 0.11)	0.709	−0.04 (−0.11; 0.04)	0.369
PC3	−0.12 (−0.18; −0.05)	<0.001	−0.12 (−0.21; −0.03)	0.008	−0.05 (−0.12; 0.03)	0.224
PC4	0.03 (−0.04; 0.10)	0.389	0.00 (−0.09; 0.10)	0.942	−0.02 (−0.10; 0.06)	0.706
PC5	0.02 (−0.05; 0.09)	0.542	0.09 (−0.01; 0.19)	0.073	0.07 (−0.01; 0.15)	0.078
PC6	0.05 (−0.01; 0.12)	0.123	0.03 (−0.07; 0.12)	0.576	0.00 (−0.08; 0.07)	0.928
PC7	−0.04 (−0.11; 0.03)	0.247	−0.10 (−0.19; −0.01)	0,035	−0.07 (−0.15; 0.00)	0,066
						
**WOMEN**						
PC1	0.04 (−0.04; 0.11)	0.349	−0.06 (−0.19; 0.07)	0.330	−0.09 (−0.21; 0.02)	0.114
PC2	0.05 (−0.02; 0.12)	0.171	−0.08 (−0.19; 0.04)	0.175	−0.12 (−0.22; −0.02)	0.024
PC3	0.00 (−0.07; 0.07)	0.948	−0.05 (−0.16; 0.06)	0.369	−0.04 (−0.14; 0.06)	0.449
PC4	−0.04 (−0.11; 0.04)	0.313	−0.05 (−0.17; 0.07)	0.405	−0.02 (−0.13; 0.09)	0.669
PC5	0.07 (0.00; 0.14)	0.037	0.14 (0.03; 0.26)	0.014	0.10 (−0.01;0.20)	0.070
PC6	0.08 (0.00; 0.15)	0.036	0.14 (0.02; 0.26)	0.023	0.07 (−0.04; 0.17)	0.237
PC7	0.03 (−0.04; 0.10)	0.436	0.03 (−0.09; 0.15)	0.585	−0.01 (−0.12; 0.10)	0.831

1 adjusted for age, baseline weight, baseline height and physical activity status (n = 552 in men, 539 in women).

2 adjusted for age, baseline height, baseline weight, baseline WC and physical activity status (n = 547 in men, 534 in women).

3 adjusted for annual change in BMI, age, baseline height, baseline weight, baseline WC and physical activity status (n = 547 in men, 532 in women).

Female PC2 and PC3 showed similar, though coefficient-reversed, patterns as male PC2 and PC3, with loadings of medium chain MUFA and n-3 LC-PUFA (positive in men, negative in women) opposing loadings of TFA (negative in men, positive in women) in PC2, and SFA and PUFA (positive in men, negative in women) opposing 18:2 TFA (negative in men, positive in women) in PC3. In men, PC2 was significantly positively associated with changes in weight (0.09 kg/yr, 95% confidence interval (CI) 0.02 to 0.15 per SD), while PC3 was significantly negatively associated with changes in weight and in WC (−0.12 kg/yr, 95% CI -0.18 to -0.05 per SD and −0.12 cm/yr, 95% CI -0.21 to -0.03 per SD, respectively). In women, PC2 was negatively associated with changes in WC_BMI_ (−0.12 cm/yr, 95% CI -0.22 to -0.02 per SD). There were no associations between changes in body size and female PC3.

Positive weights of LC-MUFA, TFA and n-3 LC-PUFA, and negative loadings of n-6 LC-PUFA in both men and women characterized PC4, and there were no associations with changes in body size.

Male and female PC5 were negatively loaded with MUFA and positively with TFA and PUFA. There were no associations between male PC5 and changes in body size. In women, PC5 was marginally positively associated with change in weight (0.07 kg/yr, 95% CI 0.00 to 0.14 per SD) and significantly positively associated with change in WC (0.14 cm/yr, 95% CI 0.03 to 0.26 per SD).

Male and female PC6 showed broad similarities in shorter chain fatty acid patterns, and positive weights of n-6 LC-PUFA. The loadings appeared specific to individual fatty acids rather than classes of fatty acid types, and weights of TFA were close to zero in women. In both men and women, PC6 was marginally positively associated with changes in weight (0.05 kg/yr, 95% CI -0.01 to 0.12 per SD in men and 0.08 kg/yr, 95% CI 0.00 to 0.15 per SD in women). In women, PC6 was also associated with changes in WC (0.14 cm/yr, 95% CI 0.02 to 0.26 per SD).

In male and female PC7, TFA loaded negatively, while 18:2 n-6 (9t12c), 18:2 n-6 and 20:2 n-6 loaded positively (Figure 2). Male PC7 was negatively associated with changes in WC (−0.10 cm/yr, 95% CI −0.19 to −0.01 per SD) and marginally negatively associated with changes in WC_BMI_ (−0.07 cm/yr, 95% CI -0.15 to 0.00 per SD).

## Discussion

In this study we observed principal components with distinct fatty acid loading patterns that may be important in the development of overweight and obesity. In men, a pattern mainly characterized by high levels of SFA and PUFA and low levels of TFA (PC3), was negatively associated with both changes in weight and in WC. Similarly, a pattern characterized by high levels of n-6 PUFA and low levels of TFA was negatively associated with changes in WC and in WC_BMI_ (PC7). In women, patterns characterized by high levels of TFA and PUFA and low levels of MUFA (PC5) or by high levels of n-6 LC-PUFA and lower levels of TFA (PC6) were positively associated with both changes in weight and in WC. No clear associations were seen for patterns with high levels of n-3 LC-PUFA.

Interpretation of our results is complicated as individual fatty acids or groupings of fatty acids that appear to stand out in a pattern did not alone determine the associations of the pattern with changes in anthropometry. The associations had to be evaluated according to the context provided by other fatty acids in the pattern. For example, the varying weights of fatty acids across patterns such as male PC2 and PC3 modified the expected associations between the high levels of n-3 LC-PUFA, present in both factors, with changes in anthropometry. Investigating associations between individual n-3 LC-PUFA, adjusted for the other fatty acids in the adipose tissue, would have obscured these effects. This context dependence may be considered a strength of our study results, as it can be used to inspire future studies of the adipose tissue fatty acids modifying the effects of fatty acids loading heavily on a pattern. Traditional investigation of effect modification using stratification or interaction terms in a regression model is not possible within factor scores. Thus alternative approaches to exploring modification may lead to new hypotheses, such as SFA modifying the effects of n-3 LC-PUFA as in PC2, that can be tested biologically. Interpreting components according to the variables with high factor loadings may therefore also be misleading and limits the exploratory nature of PCA studies. However, this approach is common [19], and was also explored here to facilitate interpretation of our results.

The proportions of fatty acids in adipose tissue biopsies from participants with a high score of a given component conform more closely to the fatty acid pattern in question than those of participants with a low score. While patterns with high levels of TFA (female PC5 and PC6) tended to be positively associated with changes in weight or WC, and conversely patterns with low levels of TFA (male PC3 and PC7) were negatively associated with changes in weight or WC, trends were not so clear for patterns with high levels of n-3 LC-PUFA. We observed a pattern with high levels of n-3 LC-PUFA (PC2) that was only positively associated with changes in weight in men. However other patterns with high levels of n-3 LC-PUFA, such as male PC3 or female PC4, were not positively associated with changes in anthropometry, so that associations with changes in anthropometry for n-3 LC-PUFA were dependent on the context of the rest of the fatty acid pattern. Our findings may indicate that men with high scores, and thus high dietary intakes of n-3 LC-PUFA or enhanced endogenous desaturation and elongation of α-linolenic acid, were more likely to gain weight than those with low scores when levels of saturated fat and CLA were low (PC2, Figure 2). But causal interpretation of these results is hampered by the nature of PCA, where every input variable contributes to each component, and subtle patterns may be obscured by the resulting “noise” [35]. This drawback to the interpretation of PCA results has also been noted for dietary studies [19], and other algorithms for determining patterns in fatty acids that are sparser may be more suited for hypothesis generation regarding primary associations in the future [35].

Selection bias is unlikely to have affected our results. The study population is a random sample of the ongoing Danish cohort study Diet, Cancer and Health, itself a sample of Danes living in the major urban and suburban areas of Denmark. A total of 337 participants (18%) in the sub-cohort were lost to follow-up. These participants were of slightly greater WC and BMI and more likely to be current smokers than those for whom follow-up data were available; however, it is unlikely that the relations between fatty acid patterns and changes in body size were different for these persons. Measurement error in fatty acid values was minimized through rigorous laboratory techniques, and missing fatty acid values were imputed using an EM algorithm [28]. Height, weight and waist circumference were measured by trained staff at baseline, and provided by self-report at follow-up. A validation study within the cohort found that these self-reported measurements can be used to investigate changes in weight if adjusting models for baseline weight and height, and models of changes in WC for baseline weight, height and WC [32], as we did. These adjustments capture potential factors associated with selective misreporting of weight and WC [36]. By assessing changes in anthropometry prospectively rather than cross-sectionally, we avoided bias due to reverse causation. It is likely that genetics and lifestyle affect metabolism and storage of fatty acids, but we were unable to assess potentially modifying effects of genetic variation. However we adjusted our analyses for physical activity, for which good evidence of effects on adipose tissue composition exists [17]. It is also possible that some of the statistically significant results we observed are due to chance, as many tests were performed.

Previous studies have not investigated adipose tissue fatty acid patterns and their relations with changes in anthropometry, however the proportions of fatty acids in adipose tissue determined in our study are similar to those reported in a comprehensive review of previous studies [17]. Some of the fatty acid patterns determined in our study population are also similar to those determined in 2 previous studies in men investigating serum fatty acid patterns, which reflect short-to medium-term dietary intake and are under greater homeostatic regulation [17]. Skidmore et al [21] reported a PC with high levels of SFA and low levels of n-6 LC-PUFA, similar to PC1 in our study; a PC with high levels of n-3 and n-6 LC-PUFA, similar to male PC5 in our study; and a PC with high levels of 18:1 n-9 and low levels of 22:4 n-6, similar to our male and female PC6 and male PC7. Warensjo et al [22] reported a PC with high levels of 16:0, 18:0 and LC-PUFA, similar to our male and female PC3; and a PC with high levels of n-3 LC-PUFA and low levels of 18:0, similar to our male and female PC4. They also reported a PC with high levels of 16:1 n-7, 18:1 n-9 and low levels of 18:2 n-6, which we were unable to find in our PCA-derived patterns. Our results also agree with some previous studies of individual fatty acids that have shown positive associations between individual n-6 LC-PUFA or TFAs, measured by dietary intake or in adipose tissue, and changes in weight [14], [15], [37].

A strength of our study lies in the assessment of fatty acid patterns, which detect information about correlations between fatty acids [21], [22], and in relating these patterns prospectively to changes in anthropometry. Analysis of fatty acid patterns may elucidate the combined effects of multiple fatty acids acting simultaneously in the body [21] - which studies of individual fatty acids could not - and are representative of the true endogenous exposure after digestion, metabolism and storage in adipose tissue. However, interpretation of the patterns themselves is not trivial. Future studies may benefit from the use of other data dimension reduction methods that produce patterns that are less complex to interpret, such as sparse PCA [38] or the treelet transform [35], [39]. Supervised methods which take into account the outcome when deriving patterns may also be of interest. Our results indicate that fatty acid patterns are associated with changes in anthropometry, but the underlying mechanisms are unclear. TFA may induce insulin resistance [8] leading to gains in adiposity, and there is evidence of gene-diet interactions with n-6 LC-PUFA intake in risk of obesity [40], [41], but further research is needed into how these effects are combined in the patterns determined here. Further research is also needed to elucidate how genetics, dietary habits and individual metabolism interplay in their contributions to adipose tissue composition. Investigation of dietary intakes reported by participants with adipose tissue fatty acid patterns positively associated with changes in anthropometry may help translate our results into public health measures.

In conclusion, we found clearly recognizable patterns of fatty acid groups in this investigation of adipose tissue fatty acids. Adipose tissue fatty acid patterns with high levels of TFA tended to be positively associated with changes in weight and in WC for both men and women. Patterns with high levels of n-6 LC-PUFA tended to be negatively associated with changes in weight and WC in men, and positively associated in women. Associations with changes in anthropometry for patterns with high levels of n-3 LC-PUFA were dependent on the context of the rest of the fatty acid pattern. These results suggest that fatty acid patterns with high levels of TFA may be linked to weight gain, but patterns with high n-3 LC-PUFA did not appear to be linked to weight loss, and that associations depend on the characteristics of the rest of the pattern. Elucidation of the simultaneous effects of fatty acids in dietary intake and in metabolism via statistically derived patterns may be important in studies of overweight and obesity.

## Supporting Information

Figure S1 **Heatmap of Pearson correlation coefficients between 34 adipose tissue fatty acids in 1100 men and women.** Δ nomenclature in parentheses indicates the location of the double bond from the carboxyl terminal. * Peaks for 18:1n-10t and 18:1n-12t, and for18:1n-6t and 18:1n-8t, could not be separated.
(EPS)

Table S1 **Factor loadings of the 7 retained principal components for men (a) and women (b).**
(DOCX)
