# Supplementary material for: Adipose Tissue Fatty Acid Patterns and Changes in Anthropometry: A Cohort Study
Source: PLoS One. 2011 Jul 21;6(7):e22587. doi: 10.1371/journal.pone.0022587 (PMC3141072; doi:10.1371/journal.pone.0022587)
Supplement: Table S1 — Factor loadings of the 7 retained principal components for men (a) and women (b). (DOCX) [file pone.0022587.s002.docx]

Table S1. Factor loadings of the 7 retained principal components for men (a) and women (b).

A.

| Fatty acid | PC1 | PC2 | PC3 | PC4 | PC5 | PC6 | PC7 |
| --- | --- | --- | --- | --- | --- | --- | --- |
| 12:0 | 0.2317 | 0.0221 | 0.0370 | 0.1096 | -0.1565 | 0.0930 | 0.2499 |
| 14:0 | 0.2436 | -0.0176 | 0.2903 | 0.0284 | -0.0736 | 0.0946 | 0.0487 |
| 15:0 | 0.2056 | -0.0825 | 0.3190 | 0.0159 | 0.0969 | -0.1069 | -0.0280 |
| 16:0 | 0.1255 | 0.0448 | 0.1830 | -0.3329 | 0.0518 | 0.2640 | -0.1582 |
| 17:0 | 0.2559 | 0.0848 | 0.1494 | -0.1857 | 0.1030 | 0.0132 | -0.0484 |
| 18:0 | 0.2415 | 0.1902 | 0.0010 | -0.2197 | -0.0902 | 0.1208 | 0.0468 |
| 19:0 | 0.2734 | 0.0079 | 0.1224 | 0.0431 | 0.0236 | 0.0352 | 0.1093 |
| 20:0 | 0.1811 | 0.1368 | -0.1467 | 0.1546 | -0.2667 | 0.2457 | 0.0128 |
| 14:1n-5 | -0.0458 | -0.2560 | 0.2446 | 0.2858 | -0.1074 | -0.0244 | 0.0278 |
| 16:1n-7 | -0.2143 | -0.2309 | 0.1196 | 0.1872 | -0.1303 | 0.0637 | -0.0877 |
| 18:1n-7 | -0.2541 | -0.1206 | -0.0722 | 0.0503 | -0.1255 | 0.2189 | -0.0828 |
| 18:1n-9 | -15380. | -0.1389 | -0.1717 | -0.0264 | -0.1886 | -0.2497 | -0.1750 |
| 20:1n-9 | -0.0735 | 0.3000 | -0.0160 | 0.2523 | -0.2438 | 0.1310 | -0.0851 |
| 20:1n-11 | 0.0169 | 0.3061 | 0.1256 | 0.2466 | -0.0971 | 0.0096 | -0.2034 |
| 22:1n-9 | 0.0171 | 0.2321 | 0.0064 | 0.1583 | -0.3483 | 0.1517 | 0.1204 |
| 16:1n-7 (∆9t) | 0.1196 | -0.1394 | -0.0459 | 0.1451 | 0.2177 | 0.0245 | -0.4157 |
| 18:1n-10+12 (∆6t+∆8t)* | 0.2005 | -0.0186 | -0.2904 | 0.1612 | 0.1335 | 0.1770 | -0.1811 |
| 18:1n-9 (∆9t) | 0.1763 | -0.0413 | -0.2900 | 0.1926 | 0.1590 | 0.1731 | -0.1792 |
| 18:1n-6+8 (∆10t+∆12t)* | 0.2241 | -0.0267 | -0.2330 | 0.1653 | 0.1678 | 0.1756 | -0.0943 |
| 18:1n-7 (∆11t) | 0.2958 | 0.0283 | 0.0520 | -0.0030 | 0.0959 | 0.0825 | 0.0169 |
| 18:2n-6 (∆9c12t) | 0.0764 | -0.2484 | 0.0806 | 0.3289 | 0.1533 | -0.0157 | 0.1291 |
| 18:2n-6 (∆9t12c) | 0.1119 | -0.1219 | 0.2118 | 0.1533 | 0.0408 | -0.0550 | 0.3597 |
| 18:2n-6 (∆9c11t) | 0.0523 | -0.2587 | 0.1896 | 0.3082 | -0.0379 | 0.0616 | -0.0402 |
| 18:2n-6 | 0.0111 | 0.1705 | -0.1900 | 0.0868 | 0.2684 | -0.2464 | 0.4157 |
| 18:3n-3 | 0.0485 | 0.1261 | -0.2238 | 0.1315 | 0.1545 | -0.2885 | 0.0721 |
| 18:3n-6 | 0.0060 | -0.0386 | -0.1185 | 0.1526 | 0.2259 | 0.0277 | 0.0647 |
| 20:2n-6 | -0.1684 | 0.1430 | -0.1497 | 0.1695 | -0.0124 | 0.2116 | 0.3327 |
| 20:3n-6 | -0.1490 | 0.0213 | 0.0170 | -0.0692 | 0.3039 | 0.3045 | 0.1471 |
| 20:4n-3 | -0.1011 | 0.3028 | 0.1380 | 0.1046 | 0.1238 | -0.0375 | -0.0548 |
| 20:4n-6 | -0.2088 | -0.0979 | 0. 0674 | -0.0755 | 0.2421 | 0.2478 | 0.0972 |
| 20:5n-3 | -0.1325 | 0.2394 | 0.2042 | 0.1364 | 0.2162 | -0.1375 | -0.1697 |
| 22:4n-6 | -0.2194 | -0.0811 | 0.0372 | -0.0877 | 0.1158 | 0.4286 | 0.1136 |
| 22:5n-3 | -0.1618 | 0.2276 | 0.2228 | 0.1026 | 0.2131 | 0.1145 | -0.0901 |
| 22:6n-3 | -0.1101 | 0.2952 | 0.2032 | 0.1578 | 0.1554 | -0.0501 | 0.-1112 |

* Peaks for 18:1n-10t and 18:1n-12t, and for18:1n-6t and 18:1n-8t, could not be separated.

** ∆ nomenclature in parentheses indicates the location of the double bond from the carboxyl terminal.

B.

| Fatty acid | PC1 | PC2 | PC3 | PC4 | PC5 | PC6 | PC7 |
| --- | --- | --- | --- | --- | --- | --- | --- |
| 12:0 | 0.2543 | 0.0193 | 0.0322 | 0.0294 | -0.0245 | 0.1482 | 0.0875 |
| 14:0 | 0.2482 | 0.0207 | -0.2498 | 0.1500 | 0.0131 | 0.1325 | 0.0301 |
| 15:0 | 0.1470 | 0.1215 | -0.2997 | 0.1899 | 0.0876 | -0.1421 | 0.0853 |
| 16:0 | 0.0722 | -0.1993 | -0.2996 | -0.0758 | 0.1438 | 0.2163 | -0.1985 |
| 17:0 | 0.2390 | -0.1627 | -0.2037 | 0.0291 | 0.1204 | -0.0169 | -0.0068 |
| 18:0 | 0.2661 | -0.2242 | -0.0645 | -0.0603 | -0.0097 | 0.1153 | -0.0617 |
| 19:0 | 0.2747 | -0.0019 | -0.0959 | 0.1070 | 0.0461 | 0.0664 | 0.0652 |
| 20:0 | 0.2413 | -0.0446 | 0.2335 | 0.0249 | -0.1060 | 0.2114 | -0.0547 |
| 14:1n-5 | -0.0353 | 0.3453 | -0.0701 | 0.2425 | -0.1645 | 0.0307 | 0.0719 |
| 16:1n-7 | -0.1721 | 0.2686 | -0.0235 | 0.1356 | -0.2240 | 0.0877 | -0.1125 |
| 18:1n-7 | -0.2332 | 0.0892 | 0.1120 | -0.0499 | -0.0222 | 0.2904 | 0.0521 |
| 18:1n-9 | -0.1243 | 0.1562 | 0.1524 | -0.1578 | -0.2495 | -0.1456 | -0.1101 |
| 20:1n-9 | 0.0325 | -0.1792 | 0.2823 | 0.2213 | -0.1921 | 0.1916 | 0.1075 |
| 20:1n-11 | 01092. | -0.1503 | 0.1747 | 0.3466 | -0.1518 | 0.0559 | -0.0333 |
| 22:1n-9 | 0.1382 | -0.1429 | 0.2215 | 0.1395 | -0.2465 | 0.2470 | 0.0871 |
| 16:1n-7 (∆9t) | 0.0771 | 0.2553 | 0.0891 | 0.1030 | 0.1868 | -0.1886 | -0.2185 |
| 18:1n-10+12 (∆6t+∆8t)* | 0.1924 | 0.1039 | 0.2813 | -0.0247 | 0.2442 | -0.0035 | -0.1758 |
| 18:1n-9 (∆9t) | 0.1603 | 0.1339 | 0.3022 | -0.0201 | 0.2404 | 0.0100 | -0.1886 |
| 18:1n-6+8 (∆10t+∆12t)* | 0.1910 | 0.1171 | 0.2445 | 0.0088 | 0.2832 | 0.0208 | -0.1537 |
| 18:1n-7 (∆11t) | 0.2803 | 0.0015 | -0.0541 | 0.0348 | 0.1785 | -0.0250 | 0.0276 |
| 18:2n-6 (∆9c12t) | 0.0186 | 0.3616 | 0.0399 | 0.1732 | 0.1444 | -0.0241 | 0.1967 |
| 18:2n-6 (∆9t12c) | 0.1074 | 0.1310 | -0.1792 | 0.1472 | 0.0120 | 0.0991 | 0.4366 |
| 18:2n-6 (∆9c11t) | 0.0488 | 0.3595 | -0.0181 | 0.2255 | -0.0040 | 0.0659 | 0.0791 |
| 18:2n-6 | -0.0207 | -0.1299 | 0.1521 | -0.0460 | 0.2323 | -0.3372 | 0.4493 |
| 18:3n-3 | 0.0348 | -0.0833 | 0.2447 | -0.0379 | 0.0571 | -0.3171 | 0.1703 |
| 18:3n-6 | 0.0055 | 0.0989 | 0.1875 | 0.1018 | 0.1391 | 0.1787 | -0.0987 |
| 20:2n-6 | -0.1252 | -0.1356 | 0.2145 | -0.0020 | 0.1530 | 0.2020 | 0.4554 |
| 20:3n-6 | -0.2143 | -0.0098 | -0.0617 | -0.0067 | 0.3641 | 0.1461 | 0.0460 |
| 20:4n-3 | -0.1526 | -0.1923 | -0.0009 | 0.2842 | 0.1312 | -0.1274 | -0.0425 |
| 20:4n-6 | -0.2311 | 0.0156 | -0.0474 | 0.0340 | 0.2278 | 0.2538 | -0.0784 |
| 20:5n-3 | -0.1250 | -0.1462 | -0.0074 | 0.3779 | 0.0041 | -0.2054 | -0.1688 |
| 22:4n-6 | -0.2362 | -0.0142 | -0.0338 | 0.0033 | 0.2763 | 0.3422 | -0.0086 |
| 22:5n-3 | -0.1846 | -0.1311 | -0.0124 | 0.3463 | 0.1348 | -0.0185 | -0.0832 |
| 22:6n-3 | -0.0957 | -0.2134 | 0.0168 | 0.3886 | 0.0264 | -0.1255 | -0.1144 |

* Peaks for 18:1n-10t and 18:1n-12t, and for18:1n-6t and 18:1n-8t, could not be separated.

** ∆ nomenclature in parentheses indicates the location of the double bond from the carboxyl terminal.
